# Endocytosis of hERG Is Clathrin-Independent and Involves Arf6

**DOI:** 10.1371/journal.pone.0085630

**Published:** 2013-12-31

**Authors:** Rucha Karnik, Melanie J. Ludlow, Nada Abuarab, Andrew J. Smith, Matthew E. L. Hardy, David J. S. Elliott, Asipu Sivaprasadarao

**Affiliations:** 1 School of Biomedical Sciences, University of Leeds, Leeds, United Kingdom; 2 Faculty of Biological Sciences, Multidisciplinary Cardiovascular Centre, University of Leeds, Leeds, United Kingdom; NHLBI, NIH, United States of America

## Abstract

The hERG potassium channel is critical for repolarisation of the cardiac action potential. Reduced expression of hERG at the plasma membrane, whether caused by hereditary mutations or drugs, results in long QT syndrome and increases the risk of ventricular arrhythmias. Thus, it is of fundamental importance to understand how the density of this channel at the plasma membrane is regulated. We used antibodies to an extracellular native or engineered epitope, in conjunction with immunofluorescence and ELISA, to investigate the mechanism of hERG endocytosis in recombinant cells and validated the findings in rat neonatal cardiac myocytes. The data reveal that this channel undergoes rapid internalisation, which is inhibited by neither dynasore, an inhibitor of dynamin, nor a dominant negative construct of Rab5a, into endosomes that are largely devoid of the transferrin receptor. These results support a clathrin-independent mechanism of endocytosis and exclude involvement of dynamin-dependent caveolin and RhoA mechanisms. In agreement, internalised hERG displayed marked overlap with glycosylphosphatidylinositol-anchored GFP, a clathrin-independent cargo. Endocytosis was significantly affected by cholesterol extraction with methyl-β-cyclodextrin and inhibition of Arf6 function with dominant negative Arf6-T27N-eGFP. Taken together, we conclude that hERG undergoes clathrin-independent endocytosis via a mechanism involving Arf6.

## Introduction

The hERG (human ether-a-go-go related gene) potassium channel (Kv11.1), encoded by the *KCNH2* gene, underlies the rapidly activating delayed rectifier K^+^ current (I_Kr_). This forms a crucial component of the repolarisation phase of the cardiac action potential and a reduction in its activity is associated with prolongation of the QT interval in the electrocardiogram (long QT syndrome 2; LQT2), which increases the risk of ventricular fibrillations and sudden death [1,2]. This aberration in the electrical activity of the heart has been identified for ~300 inherited mutations [2,3] and linked to a wide range of drugs [4,5], leading to their removal from the market and failure of new drugs in preclinical testing. Loss of function results from reducing the activity and/or the cell surface density of hERG. 

Surface levels are determined by the balance between channel insertion into the cell membrane, from forward (biosynthetic) trafficking and recycling of endocytic channels back to the surface, and channel removal by endocytosis. Reducing forward trafficking represents one mechanism by which hERG surface density is decreased. LQT2 mutations and drugs can cause misfolding of newly synthesised channels, resulting in their retention in the ER, polyubiquitination and degradation by the cytosolic proteasomes [6,7]. Alternatively, endocytic trafficking of hERG can be disrupted, altering channel removal from the surface, recycling back to the cell membrane and/or targeting for endosomal degradation. This mechanism is less established, but has been implicated in the impact of certain drugs [8,9] and pathophysiological conditions such as hypokalaemia [10,11] and hyperglycaemia [12,13]. Therefore it is important that we understand the fate of hERG after it is inserted in the plasma membrane, something that has so far received little attention.

Most membrane proteins are removed from the surface by endocytosis and are then either recycled back to the plasma membrane or undergo degradation [14,15]. Unlike biosynthetic delivery, which is slow (hours) [16,17], endosomal trafficking events can occur on a rapid time scale (minutes) [18,19]. Thus, a cell can adjust the surface density of a given membrane protein more readily by modifying endosomal trafficking events than by biosynthetic delivery. Endocytosis incorporates more than one mechanism for the selective removal of proteins from the cell surface, primarily categorised by the involvement of clathrin-coated pits. Clathrin-mediated endocytosis (CME) represents a single extensively studied mechanism [20] but clathrin-independent endocytosis (CIE) comprises multiple different mechanisms, with distinct dependencies on, for example, dynamin, RhoA, cdc42, Arf6 and caveolins [14]. CIE mechanisms are less well defined but appear to share a common requirement for free cholesterol in the plasma membrane [21,22]. Most internalised proteins are delivered to sorting centres, for example early endosomes (EE) and the endocytic recycling compartment. From there they are targeted for recycling, enabling cells to restore activities, selectively returning proteins to the plasma membrane, or for degradation, allowing cells to terminate signals over a longer time scale [14,15]. Key regulators of endocytic trafficking are the Rab and ADP-ribosylation factor (Arf) subfamilies of small GTPases. They act as molecular switches, involved in vesicle formation, movement and tethering and membrane fusion, by recruiting/interacting with ‘effector’ proteins [14,23].

Using a combination of cell biological, functional and biochemical approaches, we demonstrate that hERG channels undergo internalisation through a dynamin-independent mechanism involving Arf6. 

## Results

**Figure 1:**
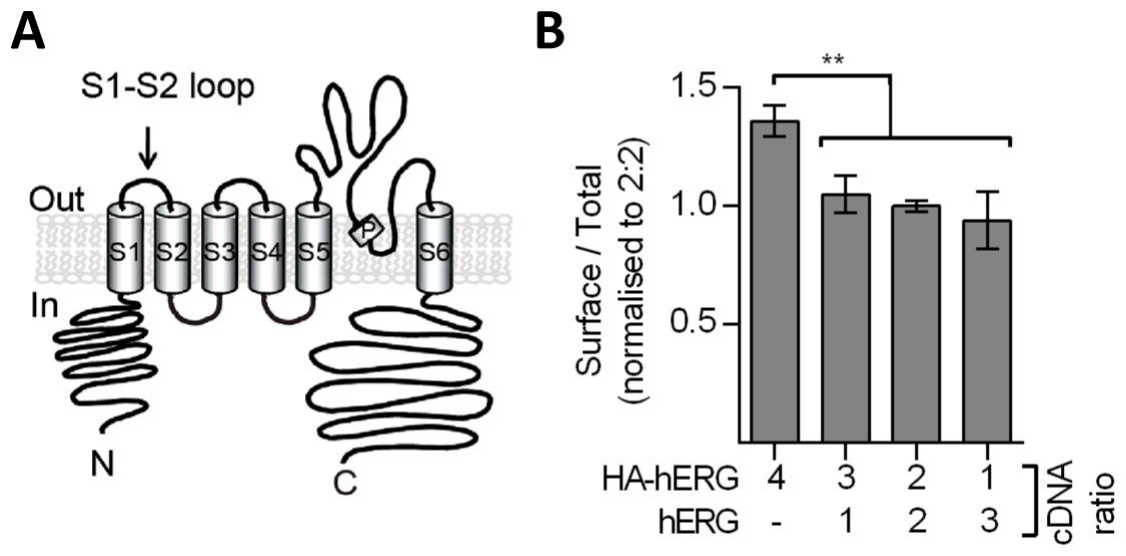
Surface density of HA-hERG containing channels. (A) Schematic of the transmembrane topology of the hERG subunit illustrating the insertion site of the HA sequence. (B) HEK-MSR cells were transfected with various ratios of HA-hERG and hERG expression vectors. Surface (non-permeabilised) and total (permeabilised) levels of HA-hERG_hERG_ were quantified using TMB substrate (n ≥ 4).

Our aim is to extend the knowledge of the fate of hERG after insertion into the cell membrane. In this study we focus specifically on endocytosis, the first step in endocytic hERG trafficking. This was achieved by using primary antibodies recognising an extracellular epitope to label channels at the cell surface and subsequently allowing them to internalise. A commercial antibody targeted against the native extracellular S1-S2 loop of hERG exists but is not suitable for use in ELISA-based quantitative assays. Therefore, the majority of the work presented utilises a hERG construct bearing a haemagglutinin A (HA) epitope engineered into the extracellular S1-S2 loop (HA-hERG) (Figure 1A) [24]. This construct has been used extensively to study hERG trafficking to the cell surface [24–27]. Antibody recognition requires only one of the four subunits of a tetrameric hERG channel to contain the HA sequence, therefore, cells were transfected with a 1:1 ratio of HA-hERG:hERG cDNA as this provided the best balance between signal strength within the assays and HA-hERG subunit inclusion (termed “HA-hERG_hERG_”). The decision to use HA-hERG_hERG_ was reinforced by the finding that HA-hERG tetramers have slightly elevated surface levels, with respect to total channel expression, in comparison with channels formed following co-expression of HA-hERG with wild type hERG; the inclusion of hERG yielded consistent surface levels irrespective of the ratio between the two constructs (Figure 1B). All findings made here were equivalent for HA-hERG and HA-hERG_hERG_ channels, suggesting that any disruption caused by the presence of four HA epitopes within the channel is only minor with respect to endocytic trafficking.

### Internalised hERG co-localises with CIE cargo

Determining whether hERG internalisation is dependent on clathrin-coated vesicles is pivotal to understanding its endocytic trafficking since intracellular targeting of CME and CIE cargoes appears to be largely distinct [19,21,28]. To identify the endocytic pathway, we first compared the endocytic distribution of hERG with established cargo markers: the transferrin (Tfn) receptor, which undergoes CME [21,29], and major histocompatibility complex class I (MHCI) and glycosylphosphatidylinositol-anchored GFP (GFP-GPI), which both undergo CIE [21,30]. 

**Figure 2:**
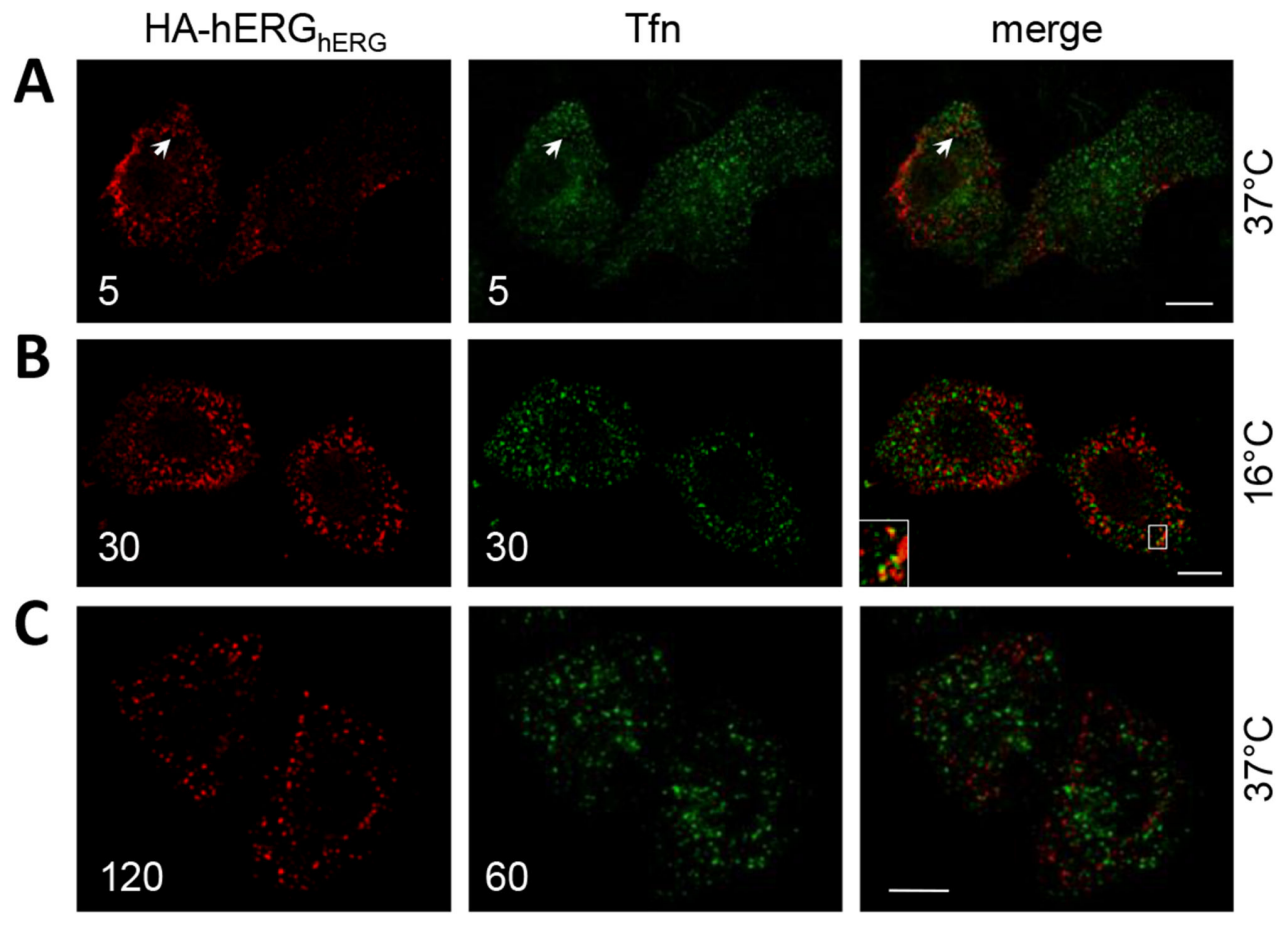
Internalised hERG channels and transferrin predominantly localise to distinct endosomes. HeLa cells transfected with HA-hERG_hERG_ were incubated with anti-HA prior to washing, fixing, permeabilisation and staining with Cy3 conjugated secondary antibody. Alexa Fluor^®^ 488-conjugated Tfn was used as marker of the Tfn receptor. Different incubation times (indicated in minutes in bottom left corner of images) at 37°C were used to compare protein distributions following 5 minutes internalisation (A) or after allowing sufficient time to label recycling pathways (C). Incubation at 16°C (B) inhibits trafficking of the Tfn receptor beyond EE, facilitating assessment of early trafficking events. Amplified boxes highlight points of co-localisation and bars = 10 µm.

HeLa cells were incubated with anti-HA and Alexa Fluor^®^ 488-Tfn to label internalising HA-hERG_hERG_ and Tfn receptors respectively. After 5 minutes at 37°C these proteins were predominately present in distinct endosomes (Figure 2A), suggesting that HA-hERG_hERG_ does not internalise through CME. After internalisation the separation of CME and CIE cargoes is normally maintained until reaching EE [28,31]. Incubation at 16°C was used to impede the progress of Tfn receptors beyond EE [31,32], facilitating comparison of trafficking up to this point. Again HA-hERG_hERG_ and Tfn staining was largely distinct (Figure 2B). Trafficking of CME and CIE cargoes can also diverge during recycling back to the cell surface [21]. The majority of Tfn receptors recycle within 60 minutes [18,33], whereas recycling of hERG channels can still be detected at 90 minutes (unpublished data). Therefore, cells were incubated with anti-HA and Alexa Fluor^®^ 488-Tfn for 2 hours and 1 hour at 37°C respectively to allow for labelling of recycling pathways. The level of co-localisation between HA-hERG_hERG_ and Tfn did not increase following this extended incubation (Figure 2C). 

**Figure 3:**
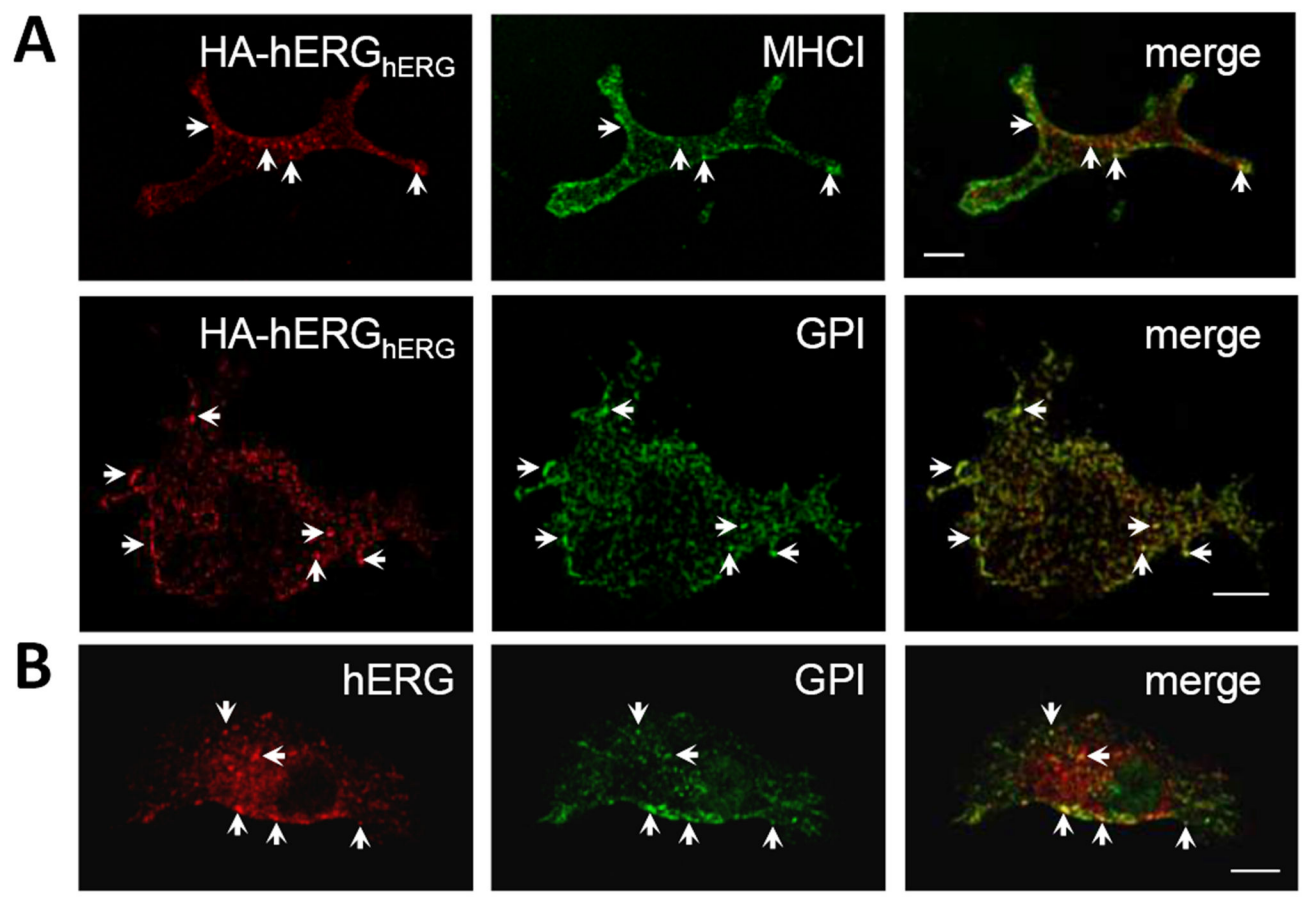
Internalised hERG channels co-localise with CIE cargo. (A) HEK-hERG cells transfected with HA-hERG and MHCI or GFP-GPI were incubated with anti-HA for 2 hours at 37°C; anti-HLA (MHCI) or anti-GFP respectively were included for the last hour. Cells were stained with Alexa Fluor^®^ 488 (MHCI) or Alexa Fluor^®^ 633 (GFP-GPI) -conjugated antibodies (both pseudo-coloured green), along with Cy3 conjugated secondary antibody (HA-hERG). (B) Internalised GFP-GPI distribution in H9c2 cells co-expressing hERG and GFP-GPI was determined as described for (A). Total hERG was stained after fixation with anti-Kv11.1 and Cy3-conjugated secondary antibody. Arrows and amplified boxes highlight points of co-localisation and bars = 10 µm.

A HEK-hERG stable cell line was transiently co-transfected with HA-hERG and MHCI or GFP-GPI. Conjointly with anti-HA, these cells were incubated at 37°C with antibodies recognising extracellular epitopes of MHCI or GFP-GPI to label the internalising CIE cargo. After allowing enough time to label both internalisation and recycling pathways [33], the results show limited co-localisation of HA-hERG_hERG_ with MHCI; instances of co-localisation were largely restricted to the cell periphery (Figure 3A). A greater degree of overlap was detected with GFP-GPI (Pearson’s coefficient ≤0.39), co-localisation being evident in both peripheral and central regions (Figure 3A). The greater similarity to the trafficking itinerary of GFP-GPI, compared with MHCI, was replicated in HeLa cells (Figure S1). Internalised GFP-GPI also showed co-localisation with hERG in H9c2 cells, a rat myoblast cell line (Figure 3B). These results further support a clathrin-independent mode of endocytosis for this channel. 

### hERG undergoes internalisation via a dynamin-independent mechanism involving Arf6 GTPase and membrane cholesterol

We next investigated the mechanism using pharmacological agents and dominant negative constructs of known mediators of endocytosis. After 30 minutes incubation with anti-HA at 37°C internalisation was assessed visually in HeLa cells and quantified in HEK-MSR cells; levels from non-permeabilised cells, representing surface channels only, were compared with those from permeabilised cells, encompassing surface plus any anti-HA-channel complexes that have internalised. Under control conditions internalised anti-HA-channel complexes represent 14.2 ± 2.2 % (n=8) of surface levels at this time point. 

**Figure 4:**
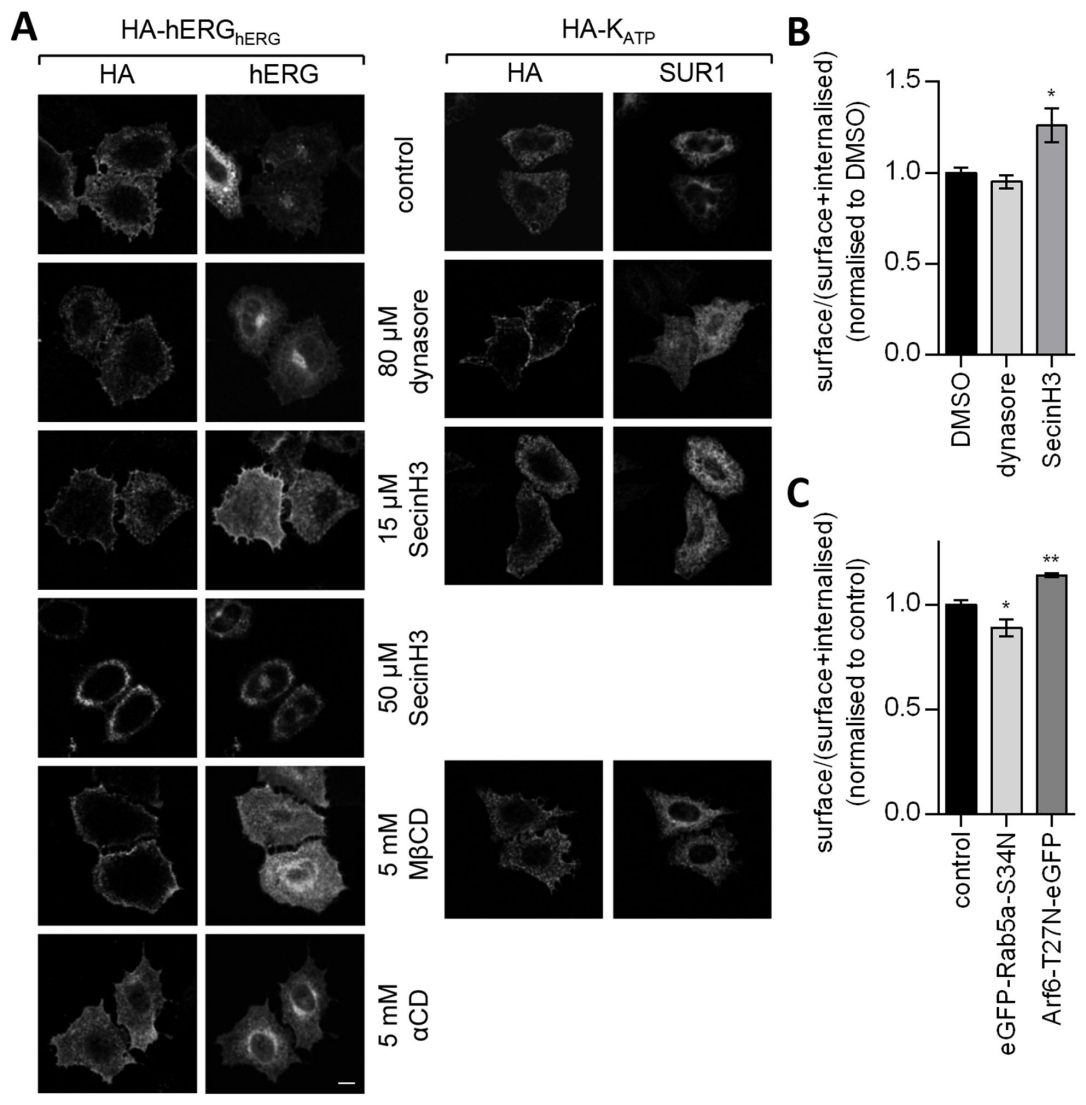
Clathrin-independent hERG internalisation. (A) The distribution of HA-hERG_hERG_ or HA-K_ATP_ transiently expressed in HeLa cells was compared after 30 minutes incubation with anti-HA in the presence or absence of 80 µM dynasore, 5 mM MβCD, 5 mM αCD or 15 µM/50 µM SecinH3. Cells were pre-incubated with each drug for 30 minutes. After fixation total HA-hERG_hERG_ or HA-K_ATP_ were stained with anti-Kv11.1 (recognising a C-terminal epitope) or anti-SUR1 respectively. Bar = 20 µm. (B-C) Internalisation of HA-hERG_hERG_ was quantified in HEK-MSR cells. After 30 minutes at 37°C with anti-HA cells were fixed, incubated with HRP-conjugated secondary antibodies and exposed to TMB substrate. Internalisation was considered as a ratio of surface (non-permeabilised cells) to surface + internalised (permeabilised cells). Increases in this ratio, indicative of decreased internalisation, were probed for after treatment with 80 µM dynasore and 15 µM SecinH3 (B) (n = 6) or co-transfection with dominant negative Rab5 and Arf6 expression vectors (C) (n = 4).

Dynasore is a chemical inhibitor of dynamin [34], a required component for CME and of certain modes of CIE [14,20]. Dynasore failed to inhibit internalisation of HA-hERG_hERG_ (Figure 4A) or alter the ratio of surface:(surface + internalised) HA-hERG_hERG_ (Figure 4B). In contrast, internalisation of HA-tagged K_ATP_, a CME cargo [35], is substantially inhibited (Figure 4A). Another protein important to CME is Rab5 [36,37]. Co-expression of eGFP-Rab5a-S34N, a GDP-locked dominant negative mutant [37], also did not increase the surface:(surface + internalised) ratio for HA-hERG_hERG_ (Figure 4C). These data further exclude CME as a mechanism for hERG internalisation. 

Most CIE mechanisms appear to depend on free cholesterol in the membrane [21,22]. Accordingly, methyl-β-cyclodextrin (MβCD), a cholesterol extracting agent [38], reduced internalisation of HA-hERG_hERG_, but not of HA-K_ATP_ channels, and α-cyclodextrin (αCD), an inactive analogue of MβCD, was without effect (Figure 4A). 

**Figure 5:**
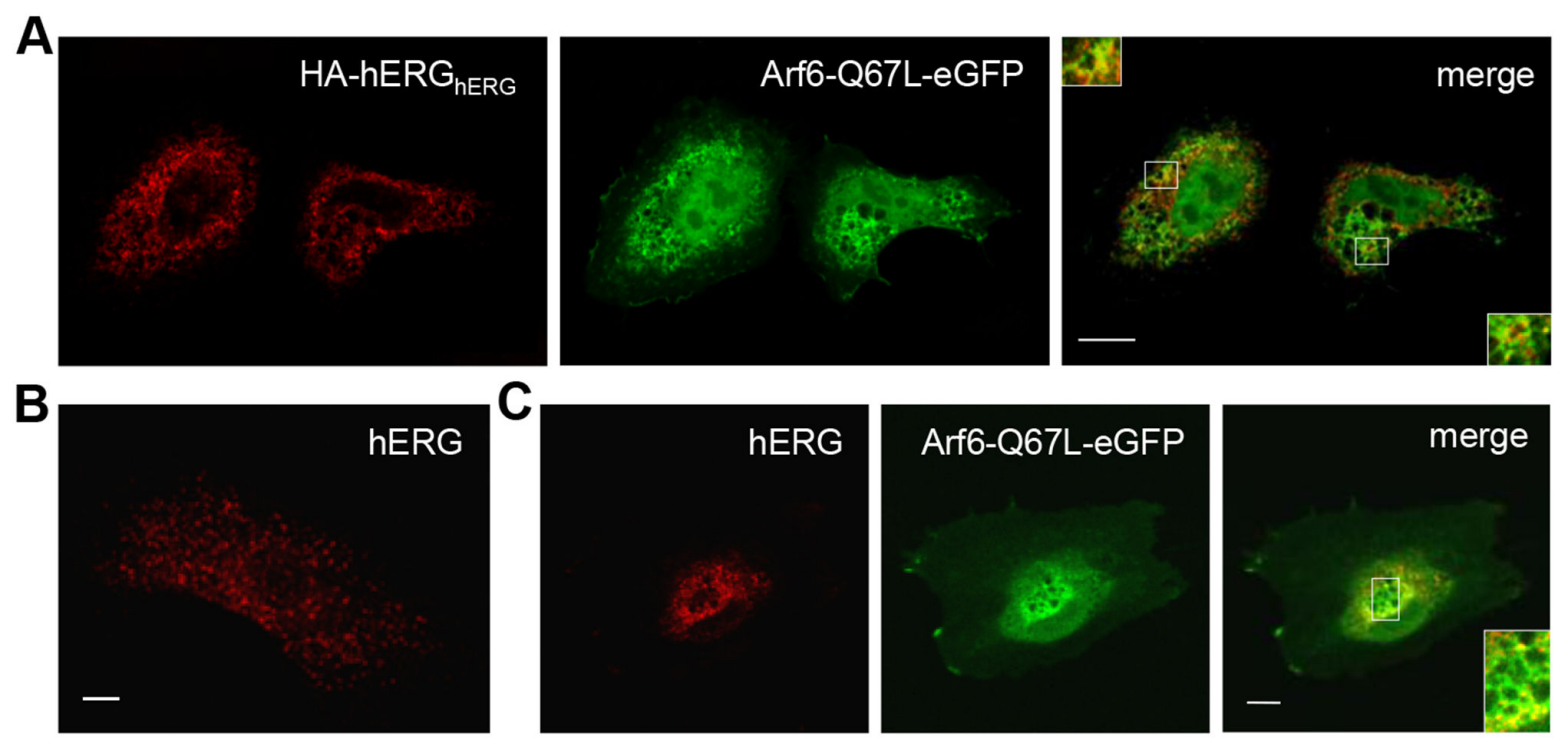
Arf6-Q67L-eGFP positive vacuoles contain hERG channels. hERG channels expressed in HeLa (A) or H9c2 cells (B&C) were stained with anti-Kv11.1 and Cy3-conjugated secondary antibodies and their distribution compared with co-expressed Arf6-Q67L-eGFP (A&C). In H9c2 cells Arf6-Q67L-eGFP (C) causes an increased central localisation of hERG compared with control (B). Amplified boxes highlight points of co-localisation and bars = 10 µm.

Irrespective of the mechanism, a common feature of CIE is internalisation of cargo into an Arf6-positive compartment. Hydrolysis of Arf6-bound GTP is required for exit from this compartment [21,28,39]. In both HeLa (Figure 5A) and H9c2 (Figure 5C) cells hERG channels were present on the characteristic vacuoles that result from expression of Arf6-Q67L, a GTPase deficient mutant [39]. Once these vacuoles accumulate within the cell, endocytosis by clathrin-independent mechanisms is greatly reduced; previously observed 30-44 hours post-transfection [39,40]. 48 hours after transfection with Arf6-Q67L-eGFP staining of HA-hERG, following 2 hours incubation with anti-HA at 37°C, is largely restricted to the cell surface (Figure S2). This staining pattern is consistent with impaired internalisation and contrasts with the extensive staining of intracellular endosomes in eGFP expressing control cells (Figure S2). 

**Figure 6:**
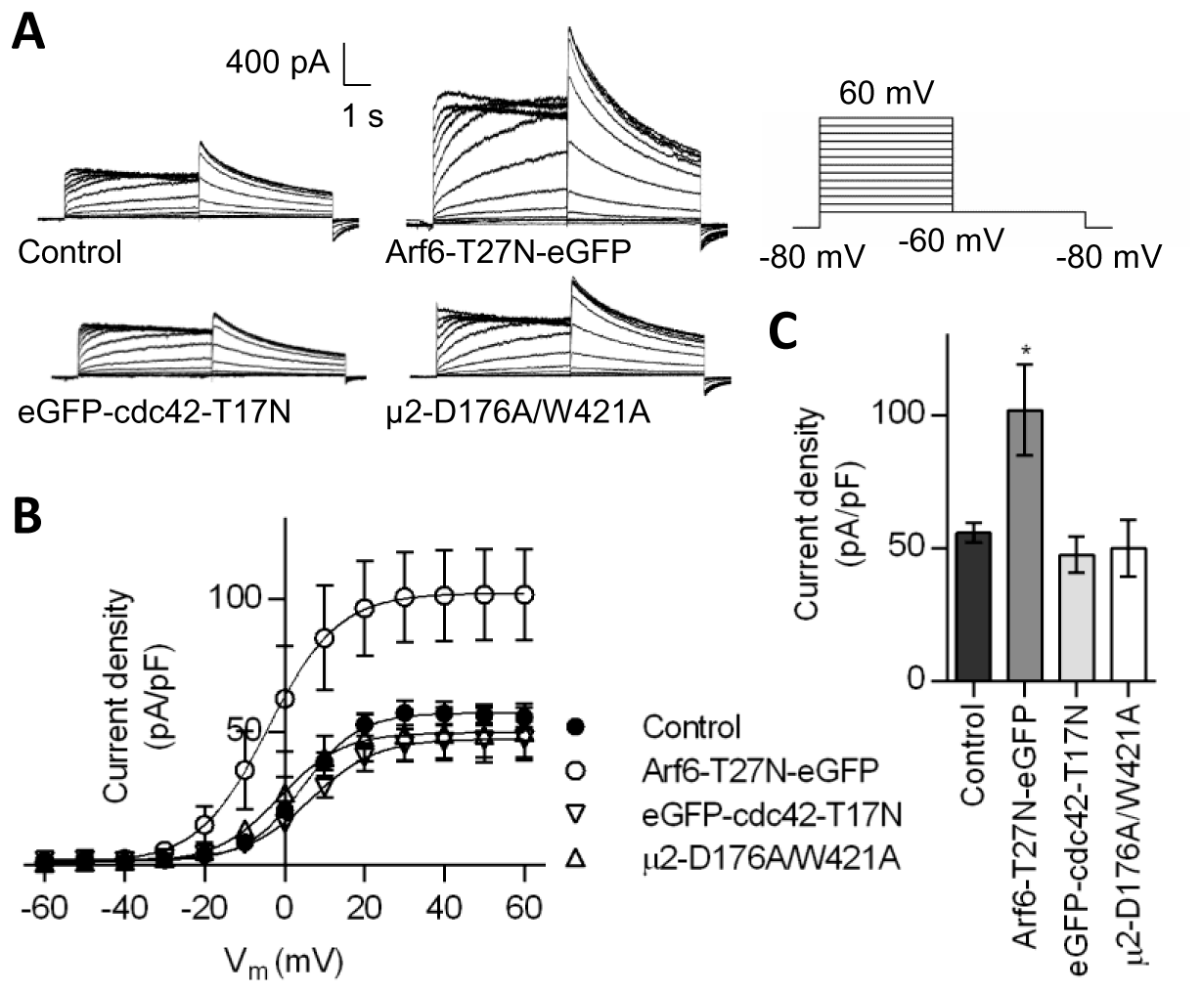
Arf6-T27N increases hERG current density. Assessment of dominant negative Arf6, cdc42 and µ2 constructs on hERG current density. (A) Current-voltage relationships of hERG channels co-expressed with Arf6-T27N-eGFP, eGFP-cdc42-T17N or µ2-D176A/W421A in HEK-MSR cells; representative current families are shown. (B) Current-voltage relationships of tail currents determined as in (A) (n = 4-9). (C) Current densities measured from tail currents at -60 mV after a +60 mV prepulse (data from (B)) (n = 4-9).

Arf1 and Arf6 are associated with dynamin-independent CIE mechanisms [41–43]. SecinH3, which inhibits cytohesins, a family of small guanine-nucleotide exchange factors for the Arf subfamily, has previously been used to investigate the role of Arf proteins in trafficking [44,45]. This compound inhibited internalisation of HA-hERG_hERG_ (Figure 4A), causing an increase in surface:(surface + internalised) ratio for HA-hERG_hERG_ (Figure 4B). Retention of HA-hERG_hERG_ at the cell surface by this drug is visible in some, but not all, HeLa cells at 15 µM and is more comprehensive at 50 µM (Figure 4A). For these cells an increase in surface level is also evident when total hERG is stained with an anti-Kv11.1 antibody recognising the C-terminus. HA-K_ATP_ is unaffected by 15 µM SecinH3 (Figure 4A). Arf1 and Arf6 negatively and positively regulate distinct mechanisms of CIE respectively [41,42]. Therefore, it is more likely that the inhibitory effect of SecinH3 on internalisation involves the latter Arf-GTPase. Accordingly, Arf6-T27N-eGFP, a dominant negative mutant [46], increased the surface:(surface + internalised) ratio for HA-hERG_hERG_ (Figure 4C), reflecting a diminished level of internalisation. An Arf6-dependent mechanism was reinforced by electrophysiological data. Arf6-T27N-eGFP increased hERG current density in HEK-MSR cells by 82.6 ± 30.5% (Figure 6). In contrast, dominant negative mutants of neither cdc42 (eGFP-cdc42-T17N), a small G-protein involved in a dynamin-independent CIE mechanism regulated by Arf1 [41,47], nor the µ2 subunit of the AP-2 clathrin adaptor subunit (µ2-D172A/W421A), which is required for Tfn receptor internalisation [48], altered hERG current density (Figure 6). 

Taken together, our results with recombinant systems indicate that hERG is internalised by CIE involving Arf6.

### ERG undergoes internalisation via a clathrin-independent mechanism involving an Arf GTPase(s) and membrane cholesterol in rat ventricular myocytes

**Figure 7:**
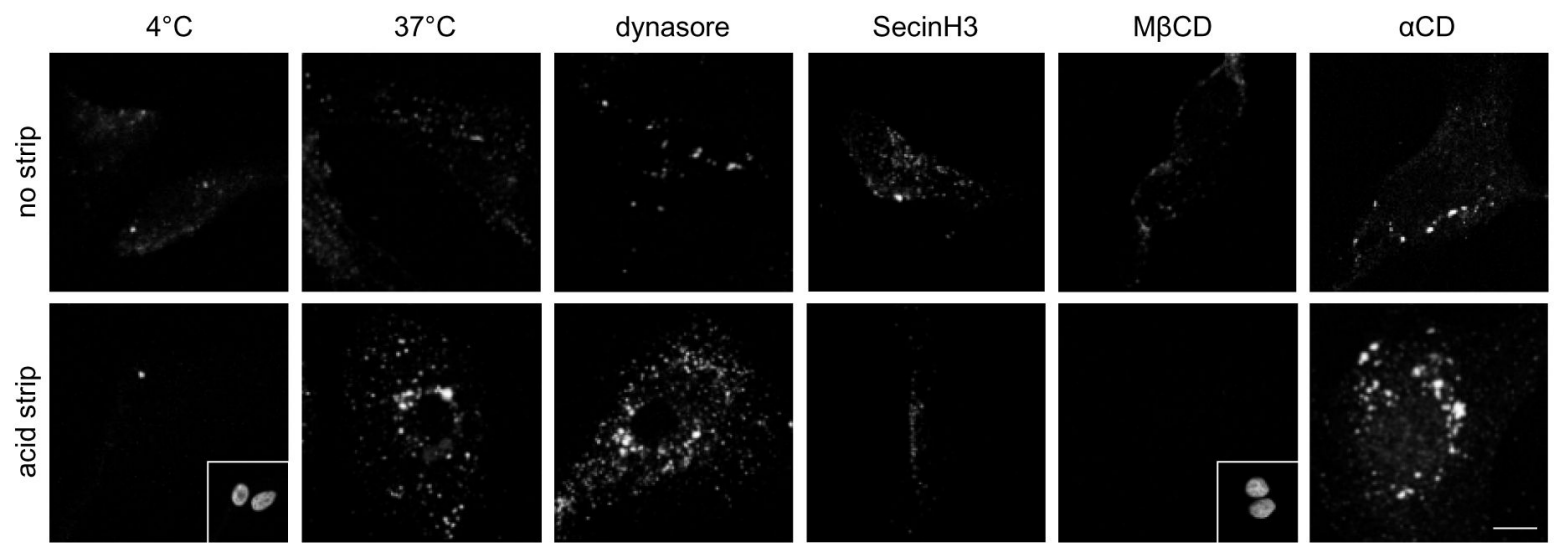
Clathrin-independent internalisation in myocytes. Effects of 80 µM dynasore, 3 mM MβCD, 3 mM αCD and 50 µM SecinH3 on internalisation of native ERG in NRVCM at 37°C. Cells were incubated for 1 hour with anti-Kv11.1 (extracellular epitope), fixed and stained with Cy3-conjugated secondary antibodies. In the bottom row, cells were washed with acidic buffer prior to fixation to remove surface bound antibodies. Staining of nuclei (inset) confirms the presence of cells within the field of view for 4°C and MβCD. Bar = 10 µm.

Finally, we sought to determine if the mechanism deduced from the recombinant system is applicable to native cells. Endocytosis of ERG in neonatal rat ventricular cardiac myocytes (NRVCM) was examined with a Kv11.1 antibody recognising the extracellular S1-S2 loop (Figure 7). At the non-permissive temperature of 4°C, Kv11.1 antibody bound to surface channels and could be stripped away by washing cells with an acidic buffer (0.5 M NaCl/0.5% acetic acid, pH 2.0). At 37°C, on the other hand, the acidic buffer wash revealed hERG present in punctate endosomes. ERG internalisation was inhibited by MβCD and SecinH3, but not by dynasore, in agreement with the data from recombinant systems using HA-hERG. 

Overall these data indicate a clathrin- and dynamin-independent but cholesterol-dependent mechanism of internalisation for hERG involving Arf6. 

## Discussion

Alterations in the plasma membrane density of hERG are associated with life-threatening cardiac arrhythmias. These changes can result from genetic mutations [2,3], a wide variety of clinically relevant drugs [4,5] and certain pathophysiological conditions, for example hypokalaemia [10,11] and hyperglycaemia [12,13]. There is growing evidence to suggest that in addition to disrupting anterograde trafficking, channel stability at the cell surface can also be targeted. To fully appreciate the mode by which hERG surface levels are modified first an understanding of the mechanism(s) that regulate them must be acquired. Plasma membrane density of most proteins is controlled by a dynamic balance between biosynthetic delivery and removal/recycling via endosomal mechanisms, the latter allowing changes to be made on a rapid time scale [18,19]. In contrast to delivery of newly synthesised hERG to the cell membrane, relatively little is known about the regulation of endosomal trafficking of this channel. It was the aim of this study to investigate the first step of this process, endocytosis. 

In all cell lines examined, including HEK-MSR, HeLa and H9c2, and in NRVCM internalisation of HA-hERG_hERG_/ERG was found to be robust and rapid. In HEK-MSR cells ~14% surface channels internalised in 30 minutes, in close agreement with previous cell surface biotinylation based results for wild type hERG (~13% in 30 minutes) [8]. By contrast, Guo and colleagues reported that internalisation of hERG occurs very slowly, taking several hours to display detectable endocytosis [11]. This inconsistency, compared with our data examining multiple cell types, potentially indicates that their stable hERG-HEK293 cell line does not provide trafficking data representative of native hERG channels. 

Most membrane proteins appear to use the classical CME to internalise proteins and ligands [20,49]. However, the number of proteins undergoing internalisation via non-conventional CIE pathways is continually increasing [14,50,51]. Examples include MHCI, GPI-AP, β1-integrin, interleukin-2 receptor-α and Kir3.4, to our knowledge the only prior example of an ion channel [21,29,52,53]. Using pharmacological and molecular approaches, we provide evidence that hERG can be added to this list.

Disruption of the activities of neither dynamin, which is required for scission of clathrin-coated vesicles [54], nor Rab5a, which regulates multiple elements of CME [55], decreased channel internalisation (Figure 4). This is in agreement with a previous observation that 6 hours incubation with dynasore, an inhibitor of dynamin, was without effect on hERG current density in HEK cells [8]. eGFP-Rab5-S34N slightly decreased the surface/(surface + internalised) ratio (Figure 4C) suggesting that channel internalisation was actually increased, an effect also occasionally noted for GPI-anchored protein uptake (CIE) in CHO cells [56]. A further indicator of CIE is the largely distinct distributions of internalised HA-hERG_hERG_ and Tfn (CME) (Figure 2), the instances of co-localisation probably corresponding to common targeting to endocytic sorting centres such as EE and the endocytic recycling compartment. Consistent with this distinction, hERG surface levels were unaffected by a dominant negative mutant of the µ2 subunit of the AP-2 clathrin adapter complex (Figure 6), which is required for recruitment of the Tfn receptor to clathrin-coated vesicles [48]. MHCI, CD59 and interleukin-2 receptor α subunit are examples of other CIE cargoes whose trafficking involves pathways divergent from those of the Tfn receptor [19,21,28]. Similar to hERG, their overlap with the Tfn receptor is limited within the first 5 mintues following internalisation and they too are present on Arf6-Q67L-eGFP enriched membranes [21,28], which are Tfn receptor negative [39]. 

A variety of regulators of CIE pathways have been identified, including RhoA, Rac1, flotillins, caveolins, cdc42, Arf1 and Arf6, but the mechanisms remain poorly defined [14]. Most CIE mechanisms share a requirement for cholesterol [21,22]. Extraction of cholesterol with MβCD indeed inhibited internalisation of HA-hERG_hERG_ in HeLa cells (Figure 4A) and also of ERG in ventricular myocytes (Figure 7). Conflicting results have been obtained regarding the presence of ERG in cholesterol and sphingolipid enriched membrane domains [57,58], so, it remains to be resolved whether hERG, like many other cardiac ion channels [59], is present in lipid rafts. Nevertheless, the lipid environment evidently impacts on both the biophysical [58] and endocytic properties of this channel in the cell membrane. 

The non-requirement for dynamin excludes caveolin- and RhoA-dependent mechanisms from amongst the CIE modes identified to date, leaving flotillin-, Arf6- and cdc42-/Arf1-related mechanisms [50]. Acute treatment with SecinH3 significantly decreased HA-hERG_hERG_ channel internalisation (Figure 4). SecinH3 inhibits cytohesins, a family of small guanine-nucleotide exchange factors for the Arf subfamily [44,45], therefore, can potentially affect both Arf1- and Arf6-related pathways. Arf1 has previously be shown to influence hERG cell surface density by an as yet undefined mechanism [60]. However, Arf1 negatively regulates cdc42-based endocytosis [41], hence, its inhibition by SecinH3 would be expected to increase, not decrease, internalisation. Also arguing against involvement of this pathway, expression of eGFP-cdc42-T17N had no effect on hERG current density (Figure 6). In contrast, Arf6-T27N-eGFP expression did reduce internalisation (Figure 4C) and increased the number of functional channels at the cell surface (Figure 6). It remains to be determined whether flotillin proteins, which are associated with a relatively slow budding rate from the plasma membrane [61], additionally contribute to hERG internalisation. 

A CIE mechanism was strengthened by demonstrating co-localisation of hERG channels with the CIE cargoes MHCI and GFP-GPI (Figures 3&S1). Although incompletely defined, the mechanisms of endocytosis of both MHCI and GPI-anchored proteins have been shown to be independent from dynamin and influenced by Arf6 activity [21,30,62,63]. After internalisation these CIE cargoes do not traffic along identical pathways [28,30], accounting for the difference in their degree of overlap with HA-hERG_hERG_. 

In contrast with the findings made here using physiological concentrations of K^+^, Zhang and colleagues reported that low extracellular [K^+^] decreases hERG cell surface density in a caveolin- and dynamin-2-dependent manner [64]. Probucol induced decreases in hERG current density are also dependent on caveolin [9] and diacylglycerol stimulated internalisation is sensitive to dynamin inhibitor peptide [65]. Therefore, while constitutive hERG internalisation is dynamin-independent, certain stimuli appear to drive its internalisation through an alternative caveolin- and/or dynamin-dependent route. 

In summary, we demonstrated that internalisation of hERG/ERG occurs via a dynamin-independent mechanism regulated by Arf6 and cholesterol. 

## Materials and Methods

### Cell lines, clones and materials

HeLa and H9c2 cell lines (obtained from Dr S Ponnambalam [66] and Prof. I Wood [67] respectively, University of Leeds) were cultured in Dulbecco’s modified Eagle medium (DMEM with GlutaMAX, Invitrogen) supplemented with 10% fetal bovine serum, 50 U/ml penicillin and 50 μg/ml streptomycin at 37°C and 5% CO_2_. GripTite^TM^ 293 MSR (HEK-MSR) (Invitrogen) and HEK-hERG (a generous gift from GlaxoSmithKline, Stevenage, UK) cell lines were maintained with 50 µg/ml and 500 µg/ml G418 respectively. Transient transfection with cDNA was carried out using FuGeneHD (Promega) or Lipofectamine 2000 (Invitrogen) 48 hours prior to performing each assay. 

Myocytes were isolated from ventricles dissected from hearts of 3-7 day old Wistar rats as described previously [68]. Briefly, cells were dissociated by incubating minced ventricles with collagenase (0.4 mg/ml, type II, Worthington) and protease (0.6 mg/ml, type XIV, Sigma) in a dissociation buffer (116 mM NaCl, 5.4 mM KCl, 0.8 mM NaH_2_PO_4_, 5.6 mM Glucose, 20 mM HEPES, 0.8 mM MgSO_4_, pH 7.35) at 37°C with agitation. Cells were filtered through a 70 μm nylon filter and cultured on glass coverslips in DMEM/F-12 medium supplemented with 10% horse serum, 5% FBS, 50 U/ml penicillin and 50 μg/ml streptomycin for 2-3 days at 37°C and 5% CO_2_. 

#### Ethics statement

All animal experimentation was carried out in accordance with the Code of Practice for the Humane Killing of Animals under Schedule 1 to the Animals (Scientific Procedures) Act 1986 and was approved by the Leeds University ethics committee: Animal Welfare and Ethical Review Committee (AWERC). Animals were humanely killed by cervical dislocation by trained personnel as per Schedule 1, a procedure that causes minimal suffering. The animals were not treated with any chemicals or subjected to experiments prior to sacrificing. 

pcDNA3-HA-hERG was produced by introducing a HA (haemagglutinin A) epitope plus an 8-amino acid linker into the S1-S2 loop of hERG [24] by polymerase chain reaction. HA-tagged Kir6.2 and SUR1 (subunits of the K_ATP_ channel) are as described [35]. The S34N mutation was introduced into Rab5a [37] by QuikChange^®^ site-directed mutagenesis (Stratagene).

Sources (in parentheses) of primary antibodies are as follows: monoclonal rat anti-HA (3F10) (Roche), affinity purified rabbit anti-Kv11.1 against the extracellular S1-S2 loop or the intracellular C-terminus (Sigma), monoclonal mouse anti-GFP and monoclonal mouse anti-HLA A2 (Abcam) and monoclonal mouse anti-SUR1 (N289/16) (NeuroMab). Sources of secondary antibodies: Cy3/Cy5-conjugated (Jackson ImmunoResearch), Alexa Fluor^®^ 488/546/633-conjugated (Invitrogen) and HRP-conjugated (Sigma). 

### Surface/total

HEK-MSR cells grown in 24 well plates were fixed with 2% PFA for 10 minutes. Surface and total HA-hERG_hERG_ were determined from non-permeabilised and permeabilised (5 minutes with 0.25% Triton X-100) wells respectively. All wells were incubated with anti-HA (100 ng/ml) followed by HRP-conjugated secondary antibodies (1:1000) in PBS-1% ovalbumin for 1 hour at room temperature. HRP activity was quantified using 3,3’5,5’Tetramethylbenzidine (TMB) (Sigma) as a peroxidase substrate. Background signal was determined from cells transfected with hERG expression vector only.

### Co-localisation using immunofluorescence

The distribution of internalised HA-hERG_hERG_ in HeLa and HEK-hERG cells was compared with different endocytic markers following up to 2 hours incubation at 37°C or 16°C with anti-HA (100 ng/ml). Endogenous transferrin receptors in HeLa cells were tracked by labelling with Alexa Fluor^®^ 488-conjugated transferrin (50 µg/ml) (Invitrogen), included for the last 5-60 minutes. Anti-HLA (0.2 µg/ml) or anti-GFP (1:500) was added for the last hour to cells co-expressing MHCI or GFP-GPI respectively. Post-fixation cells were incubated with fluorescently labelled secondary antibodies in PBS-1% ovalbumin for 1 hour at room temperature. Total hERG distribution was stained with anti-Kv11.1 targeted to the C-terminus (1:1000); performed 24 hours post-transfection for cells co-expressing Arf6-Q67L-eGFP. Stained cells were imaged on a Zeiss 510 META laser scanning confocal microscope under a 63x oil immersion lens (NA 1.40). The section thickness of all images is 1 µm. Co-localisation was assessed using ImageJ software (National Institutes of Health [69]).

### Internalisation

HeLa cells grown on coverslips were transfected with HA-hERG and hERG (1:1) or HA-K_ATP_ (HA-Kir6.2 and SUR1) plasmid constructs and subjected to an internalisation assay essentially as described previously [70]. Briefly, cells were allowed to internalise anti-HA (100 ng/ml) in DMEM/F-12-1% ovalbumin at 37°C for 30 minutes, fixed with 2% PFA for 10 minutes, permeabilised with 0.25% Triton X-100 for 5 minutes and stained with Cy3-conjugated secondary antibodies (2.4 µg/ml). Total channel distribution was stained with anti-Kv11.1 targeted to the C-terminus (1:1000) or anti-SUR1 (1:500) and respective Alexa Fluor^®^488-conjugated secondary antibodies (4 µg/ml) in PBS-1% ovalbumin for 1 hour at room temperature. Cells were pre-incubated with dynasore (Santa Cruz), SecinH3 (Calbiochem), MβCD (Sigma) or αCD (Sigma) for 30 minutes prior to addition of anti-HA plus drug. To determine endocytosis in NRVCM, cells were incubated with an anti-Kv11.1 (1:50) targeted to the S1-S2 loop of hERG for 1 hour at 37°C and washed twice with an acidic strip buffer (0.5 M NaCl/0.5% acetic acid, pH 2.0) to remove non-internalised antibody before being fixed, permeabilised and stained with fluorescent secondary antibodies. Internalisation was quantified in HEK-MSR cells using HRP-conjugated secondary antibodies and 3,3’5,5’Tetramethylbenzidine (TMB) (Sigma) as a peroxidase substrate. Surface plus internalised anti-HA-channel complex levels, from permeabilised cells, were compared with surface only levels, from non-permeabilised cells. Background signal was determined from cells transfected with hERG expression vector only and eGFP was used as a control for eGFP-Rab/Arf-eGFP constructs.

### Electrophysiology

HEK-MSR cells were co-transfected with pcDNA3-hERG and appropriate constructs. Borosilicate patch pipettes (2-3 MΩ resistance) were filled with a recording solution; 120 mM KCl, 5 mM MgCl_2_, 5 mM K_2_ATP, 5 mM EGTA and 10 mM HEPES, pH 7.2 with KOH. The bath solution contained 137 mM NaCl, 4 mM KCl, 1.8 mM CaCl_2_, 1 mM MgCl_2_, 10 mM glucose and 10 mM HEPES, pH 7.4 with NaOH. Whole currents were recorded at room temperature using an EPC10 patch clamp amplifier under the control of Patchmaster software (HEKA Electronik). Cells were held at -80 mV and 5 seconds depolarising pulses from -60 to +60 mV followed by a 5 seconds step to -60 mV were applied every 20 seconds. Current-voltage relationships were determined by plotting initial amplitudes of the tail currents at -60 mV against the applied voltage. The data were analysed using Fitmaster (HEKA) and Origin 7.0 software. 

### Data analysis

All data are presented as mean ± s.e.m. Comparison of the difference between experimental groups was made by ANOVA with Dunnett’s post-hoc test using GraphPad Prism 6.0 software. Significant differences are denoted by * (p < 0.05), ** (p < 0.01) and *** (p < 0.001). All data points originate from at least two independent experiments.

## Supporting Information

Figure S1 **Internalised hERG channels co-localise with CIE cargo.** HeLa cells transfected with HA-hERG and MHCI or GFP-GPI were incubated with anti-HA for 2 hours at 37°C; anti-HLA (MHCI) or anti-GFP respectively were included for the last hour. Cells were stained with Alexa Fluor^®^ 488 (MHCI) or Alexa Fluor^®^ 633 (GFP-GPI) -conjugated antibodies (both pseudo-coloured green), along with Cy3 conjugated secondary antibody (HA-hERG). Arrows highlight points of co-localisation and bars = 10 µm.
(TIF)

Figure S2 **Arf6-Q67L-eGFP blocks HA-hERG internalisation.** 48 hours after co-transfecting with HA-hERG and eGFP (control) or Arf6-Q67L-eGFP HeLa cells were incubated with anti-HA for 2 hours at 37°C. Permeabilised cells were stained with Cy3-conjugated secondary antibodies. Inset boxes show GFP fluorescence, confirming expression of eGFP or Arf6-Q67L-eGFP. Bars = 10 µm.
(TIF)
