# Supplementary figures and images for: Endocytosis of hERG Is Clathrin-Independent and Involves Arf6
Source: PLoS One. 2013 Dec 31;8(12):e85630. doi: 10.1371/journal.pone.0085630 (PMC3877390; doi:10.1371/journal.pone.0085630)

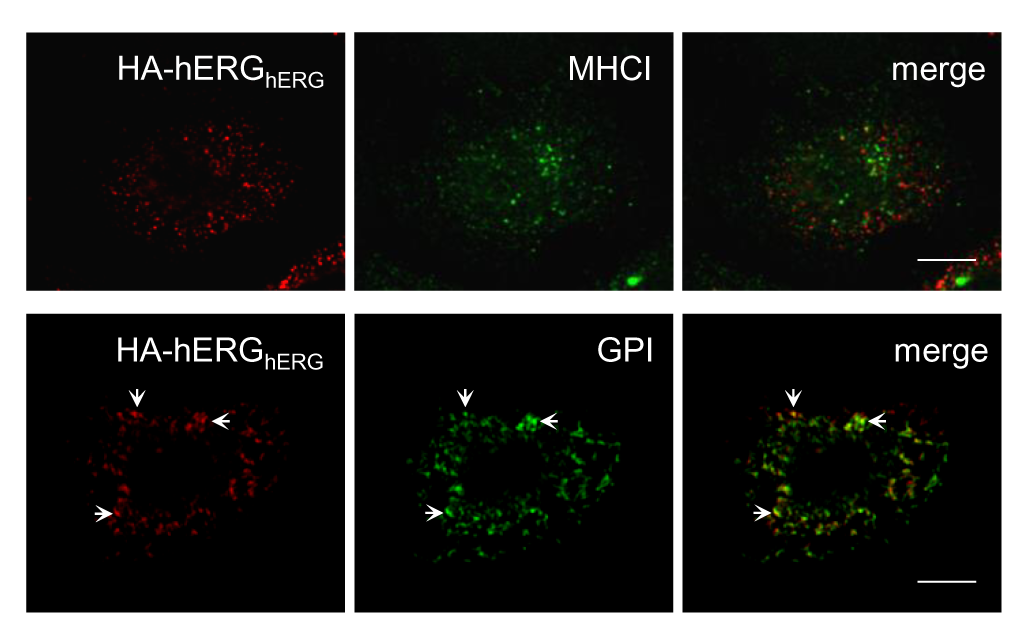

Supplement: Figure S1 — Internalised hERG channels co-localise with CIE cargo. HeLa cells transfected with HA-hERG and MHCI or GFP-GPI were incubated with anti-HA for 2 hours at 37°C; anti-HLA (MHCI) or anti-GFP respectively were included for the last hour. Cells were stained with Alexa Fluor® 488 (MHCI) or Alexa Fluor® 633 (GFP-GPI) -conjugated antibodies (both pseudo-coloured green), along with Cy3 conjugated secondary antibody (HA-hERG). Arrows highlight points of co-localisation and bars = 10 µm. (TIF) [file pone.0085630.s001.tif]

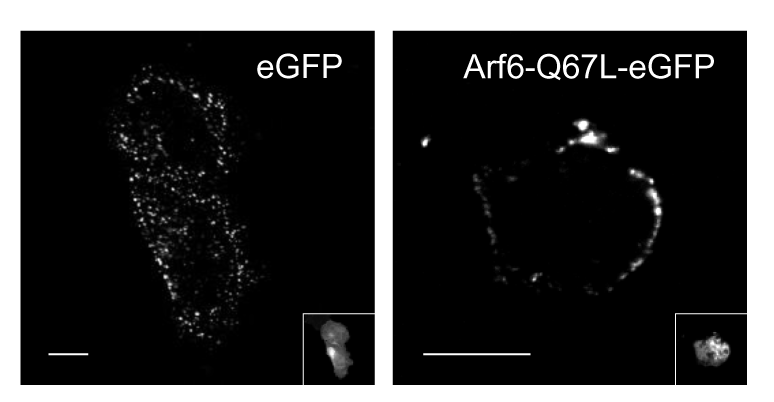

Supplement: Figure S2 — Arf6-Q67L-eGFP blocks HA-hERG internalisation. 48 hours after co-transfecting with HA-hERG and eGFP (control) or Arf6-Q67L-eGFP HeLa cells were incubated with anti-HA for 2 hours at 37°C. Permeabilised cells were stained with Cy3-conjugated secondary antibodies. Inset boxes show GFP fluorescence, confirming expression of eGFP or Arf6-Q67L-eGFP. Bars = 10 µm. (TIF) [file pone.0085630.s002.tif]
